# Mucosal Injury During Per-oral Endoscopic Myotomy: A Single-Center Experience

**DOI:** 10.5152/tjg.2022.21949

**Published:** 2022-11-01

**Authors:** Abdullah Özgür Yeniova, In Kyung Yoo, Joo Young Cho

**Affiliations:** 1 Division of Gastroenterology Tokat, Department of Internal Medicine, Tokat Gaziosmanpaşa University Faculty of Medicine, Turkey; 2 Department of Gastroenterology, Cha Bundang Medical Center, Cha University College of Medicine, Seongnam-si, Korea; 3 Department of Gastroenterology, Cha Gangnam Medical Center, Cha University College of Medicine, Gangnam-gu, Seoul, Korea

**Keywords:** Adverse event, complication, esophageal achalasia, mucosal injury, per oral endoscopic myotomy

## Abstract

**Background:**

Peroral endoscopic myotomy is associated with a low risk of adverse events. Mucosal injury is the most common intra-procedural adverse event of peroral endoscopic myotomy. Severe mucosal injury may cause serious consequences, such as esophageal leak and mediastinitis, which affect the outcome of the procedure and prolong hospital stay. The aim of the present study was to determine the characteristics, predictors, and management approaches for unintended mucosal injury during peroral endoscopic myotomy.

**Methods:**

A total of 211 patients who underwent peroral endoscopic myotomy between November 2014 and June 2019 were enrolled in this study. Mucosal injury was defined according to a previous study and maintained in the endoscopy database. Patient-related and procedure-related factors were compared between patients with and without mucosal injury. Multivariate analysis was performed after adjusting for confounding factors.

**Results:**

A total of 206 patients were eligible for study enrollment. Of these, 44 experienced mucosal injury, with an overall frequency of 21.4% (44/206). On multivariable analysis, mucosal injury was associated with submucosal fibrosis (odds ratio, 8.33; *P *= .024), intra-procedural bleeding (OR, 14.29; *P *< .001), endoscopic diameter of 9.9 mm (OR, 4.389; *P *= .006), and procedure duration over 60 minutes (OR, 1.016; *P = *.034).

**Conclusion:**

Mucosal injury is a significant event encountered during peroral endoscopic myotomy, affecting its short- and long-term outcomes. Intra-procedural bleeding, endoscopic submucosal fibrosis, and use of an endoscope with a large outer diameter have been found to be significant predictors of mucosal injury. Endoscopists should pay more attention to risk factors associated with mucosal injury to avoid adverse events.

**Main Points**

- Since per oral endoscopic myotomy (POEM) is a novel method for treatment of achalasia, data regarding its adverse events (AEs) are sparse.
- Mucosal injury (MI) is the most common adverse event encountered during POEM. There are not many studies that highlight the predictors of MI.
- Endoscopists who perform POEM should be aware of MI, its consequences, and its management since it may cause serious problems like mediastinitis.
- The present study found that submucosal fibrosis (SMF), intra-procedural bleeding (IPB), endoscopic diameter of 9.9 mm, and procedure duration over 60 minutes are predictors of MI.
- The present study is one of the few studies that outline the predictors of MI.

## Introduction

Per oral endoscopic myotomy (POEM) has gained popularity as a treatment for esophageal motility disorders worldwide since it was developed a decade ago.^1^ Accumulated data have confirmed that POEM is a safe, effective, and minimally invasive treatment for achalasia with a low rate of serious adverse events (AEs).^2^ Although POEM has emerged as a novel technique, there are sparse data to date regarding the definition, incidence, and classification of AEs attributed to the procedure. Previous studies have reported a wide range of the overall AE incidence rate (3.2%-23%) depending on the definition of AE, the classification system used to report severity, time of occurrence of the AE, and study characteristics.^3-5^

Mucosal injury (MI) is one of the most common intra-procedural events that affect the short-term outcomes of POEM. It disrupts the only barrier between the mediastinum and esophageal lumen, leading to mediastinitis. Previous studies have evaluated patient-related and intra-procedural factors that lead to the occurrence of AE^6^ but few studies have focused on factors that increase the incidence of MI. The reported incidence of MI ranges from 1.6% to 25.8%.^7^ The first study that comprehensively analyzed the predictors of MI found that patients with previous POEM and/or Heller myotomy (HM), submucosal fibrosis (SMF), mucosal edema, and tunnel length >13 cm were more vulnerable to MI.^7^ The aim of this study was to determine the characteristics, predictors, and management approaches for unintentional MI during POEM.

## Materials and Methods

Patients who underwent POEM between November 2014 and June 2019 were enrolled in this study. The present study was conducted in accordance with the Declaration of Helsinki and was approved by the Ethic Committee of CHA University Bundang Medical Center registration number: CHAMC (2015-07-115-025). Written informed consent was obtained from all of the patients before the procedure. Patients who did not provide informed consent were excluded from the study.

Procedure- and patient-related data were extracted from a prospectively recorded central database of the endoscopy unit and entered into another database developed for this study. Major AEs were defined according to a previous study that suggested a novel classification of AEs encountered during POEM (Table 1).^6^

**Figure 1.**
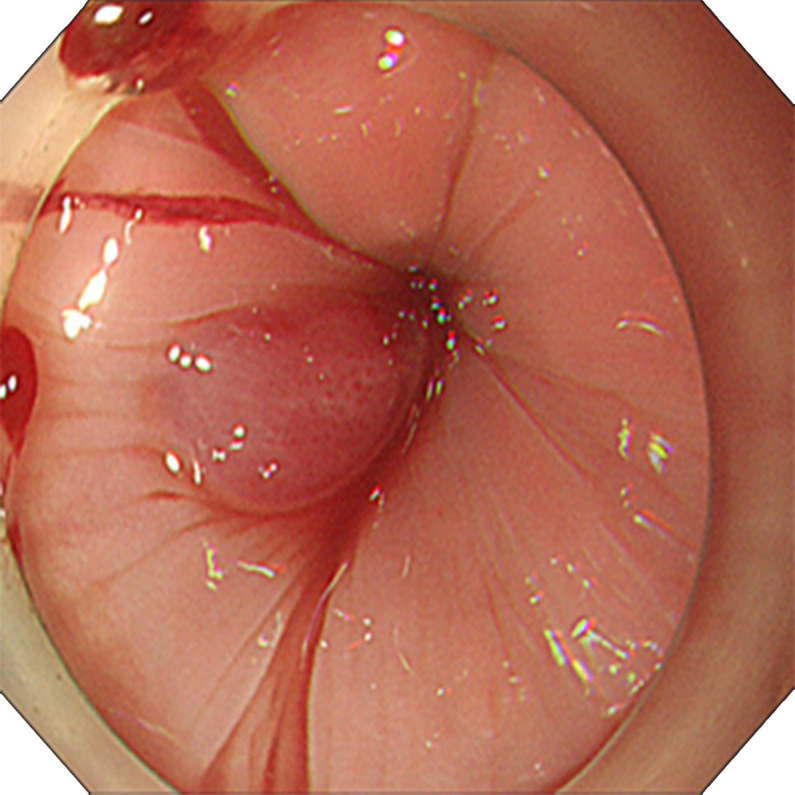
Scald mucosal injury.

**Figure 2.**
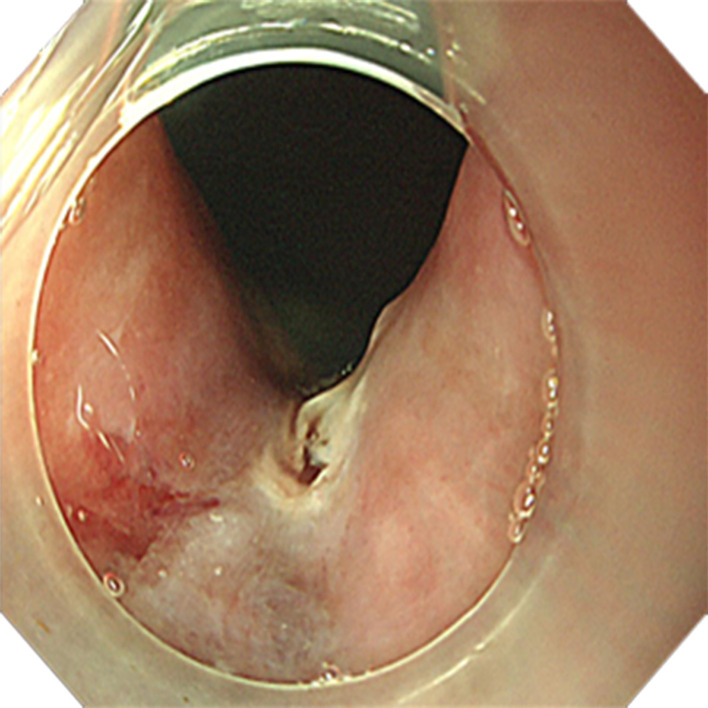
Submucosal exposure lesion (SEL).

**Figure 3.**
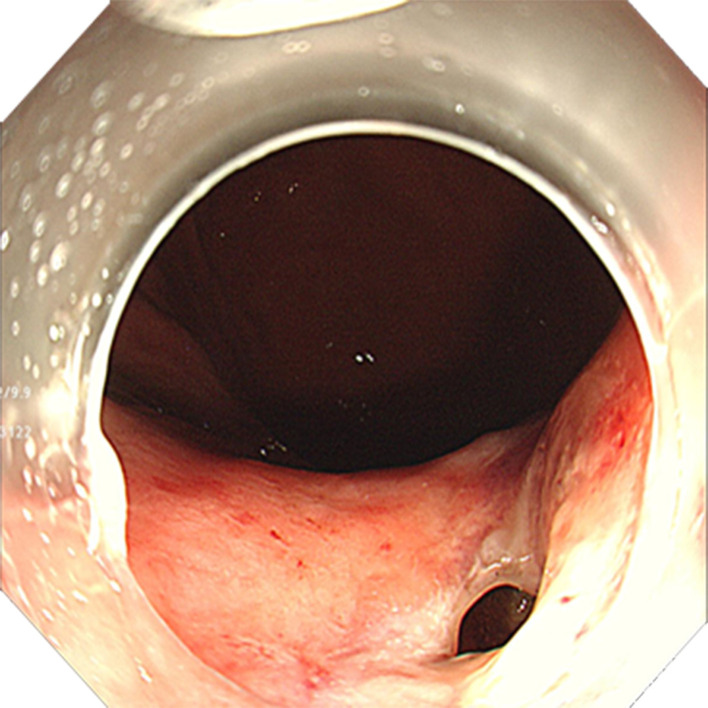
Perforation.

The MI classification system described in a previous study was used to determine MI.^7^ This study suggested a classification of MI based on clinical observations and image analyses. Three types of mucosal lesions were described in this study: scald MI (Figure 1), submucosal exposure lesion (SEL) (Figure 2), and “erforation (Figure 3) (Table 1).

The patients’ videos and photographs taken during the procedure were also analyzed by an independent researcher to retrospectively identify patients who experienced MI. Patients with scald lesions, SELs, and perforations were included in the MI group. Patients without any MI comprised the “no MI” group. Patient- and procedure-related factors were compared between the 2 groups.

Per oral endoscopic myotomy was performed as previously described.^1^ All procedures were performed by an experienced endoscopist (J.Y.C.) who has an experience of over 10 years and performs 80 cases per year under general anesthesia in the operating theater. A standard POEM technique consisting of 4 sequential steps was used for all of the patients: mucosal entry, submucosal tunnel creation, myotomy, and closure of mucosal incision.

Standard instrumentation for third-space endoscopy was used. Details on the equipment used for the procedure and other details regarding electrocautery can be seen in Table 2. Patients received peri-procedural antibiotics for prophylaxis of infection. All of the patients were followed up and fasted for at least 1 night. Patients who did not have any complaints such as hematemesis, serious chest pain, fever, or any hemodynamic instability, were allowed to consume clear liquid foods. Patients were assessed with barium esophagography, and if there was no esophageal leak or no sign of any AE, patients were discharged with proton pump inhibitors. Second-look endoscopy, chest and abdomen tomography, and blood tests were part of the initial work-up for patients who were deemed to have developed any AE. Endoscopic interventions, open or laparoscopic surgery, ICU admission, prolonged fasting, and anti-biotherapy were applied to patients where appropriate.

All 3 types of MI were repaired during POEM. Endoscopic clips (endoclips) were the main treatment tool. If closure with clips did not satisfy the endoscopist, endo-loop, fibrin glue, and over-the-scope clip (OTSC) were included in the repair process.

### Statistical Analysis

Mean and standard deviation (SD) were used for normally distributed continuous variables, and median and interquartile range for skewed data. Categorical variables have been expressed as percentages. Risk factors between groups were compared using Student’s *t*-test, Mann–Whitney *U*-test, Kruskal–Wallis test, one-way analysis of variance, and Fisher’s exact test where appropriate. Risk factors that reached a *P* value of .1 in univariate analysis were included in the multivariate analysis. Logistic regression analysis was used to calculate the 95% CIs and adjusted odds ratios (aORs). Statistical significance was set at *P *< .05. Statistical analyses were performed using Statistical Package for the Social Sciences for Windows version 24.0 (IBM Corp.; Armonk, NY, USA).

## Results

**Figure 4.**
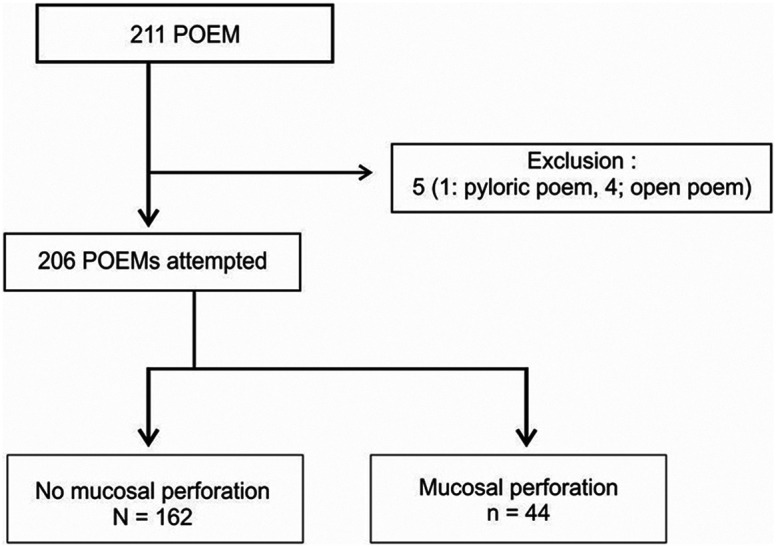
Study flow chart.

A total of 211 patients underwent POEM between 2014 and 2019. One patient who underwent gastric pyloric POEM and 4 patients who underwent open POEM were excluded from the study.^8^ The study flow diagram is shown in Figure 4.

The patients’ demographic and procedure-related factors are shown in Tables 2 and 3. Of these 206 patients, 108 (52.4%) were female and 98 (47.6%) were male. Two patients’ procedures were terminated because of severe fibrosis and converted into the open POEM procedure.

There were no significant differences in mean of age, and sex, and mean disease duration between the MI and no-MI groups. The achalasia type was shown not to have an effect on MI. There was no difference in mean of achalasia type between both groups. Patients in the MI group underwent more interventions than those in the no-MI group. The rate of MI group patients who underwent prior Botulismus toxin injection (BTI) was significantly higher than that of patients in the no-MI group. (22.7% vs 9.3%, *P* < .05). Other prior interventions, especially Heller myotomy (HM) and POEM, did not differ between the 2 groups according to our study data*. *There was no significant difference between the no-MI and the MI groups in terms of sigmoid esophagus (SE), while patients with SMF were significantly more in the MI group than in the no-MI group (18.2% vs 8.6%, *P = *.067*; *15.9% vs 1.2%, *P < *.001, respectively).

The patients’ Eckard scores, cross-sectional area (CSA), and distensibility index (DI) measurements taken before the procedure did not differ between the 2 groups. The integrated relaxation pressure (IRP) score of the no-MI group was significantly higher than that of the MI group (27.9 vs 22.3, respectively; *P = *.047).

Intra-procedural bleeding (IPB) occurred in 40 (19.4%) patients. All of the IPB cases were minor, and no patient needed a blood transfusion. Most of them were self-limiting, but 2 patients were treated with endoclips. There was a significant difference between the no-MI and the MI groups in terms of IPB. Specifically, 23 (52.3%) patients in the MI group experienced IPB, while IPB occurred in 17 (10.5%) patients of the no-MI group (*P *= .0149). There was no significant difference between the 2 groups in terms of the rate of full-thickness myotomy, myotomy length, direction of myotomy, and submucosal tunnel length. The procedure duration was significantly higher in the MI group than in the no-MI group (88.9 min vs 66.5 min, respectively; *P *< .001).

The occurrence of MI was reported in 44 (21.4%) of 206 patients who underwent POEM. Of the 44 patients, 36 (81.8%) experienced MI in the cardia region, whereas 8 patients (18.2%) had MI in the esophagus. The most common type of MI reported was scald lesions; 21 of the 44 patients (47.7%) with MI lesions were recorded as “Scald.” SEL was the second-most-common type of MI; 15 (34.1%) had SELs. The occurrence of perforations was the least common type of MI, which 8 patients (18.2%) experienced during POEM (Table 4).

**Figure 5.**
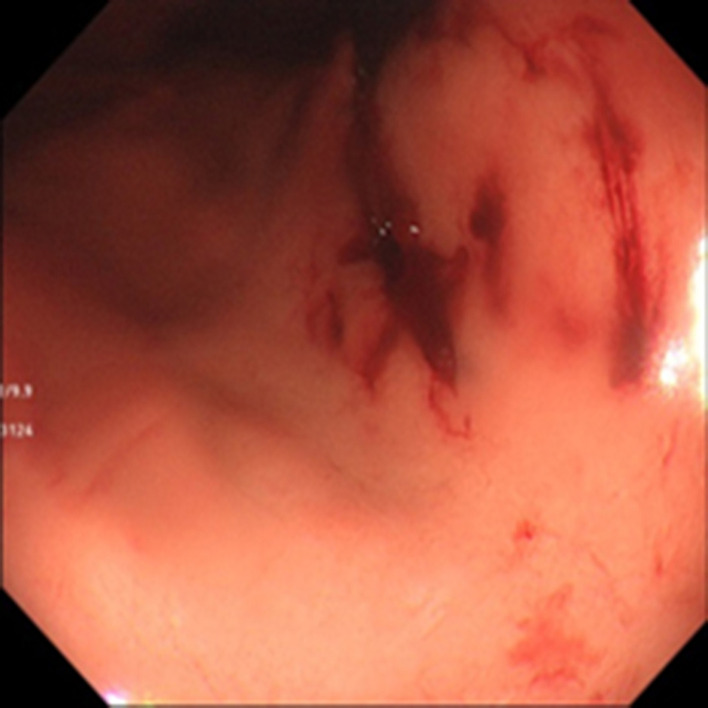
Esophagogastric junction outflow obstructrion patient with no history of previous treatment, submucosal exposure lesion in cardia.

**Figure 6.**
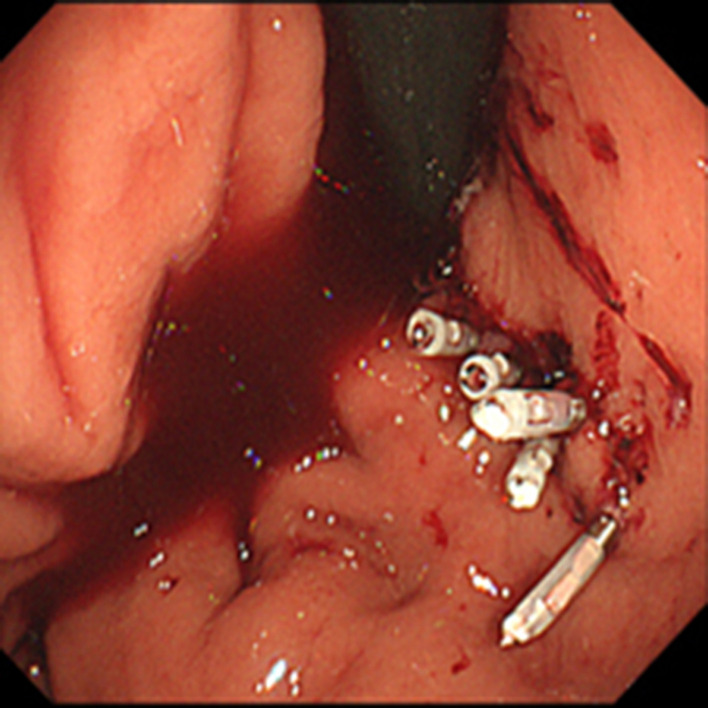
Repairment of the submucosal exposure lesion with clips.

**Figure 7.**
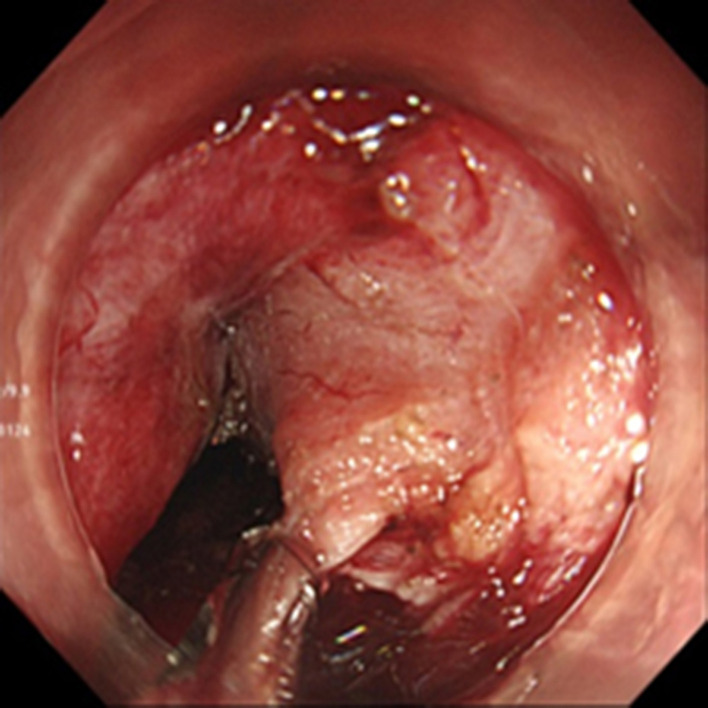
Achalasia type 2 patient with no history of previous treatment, perforation in cardia.

**Figure 8.**
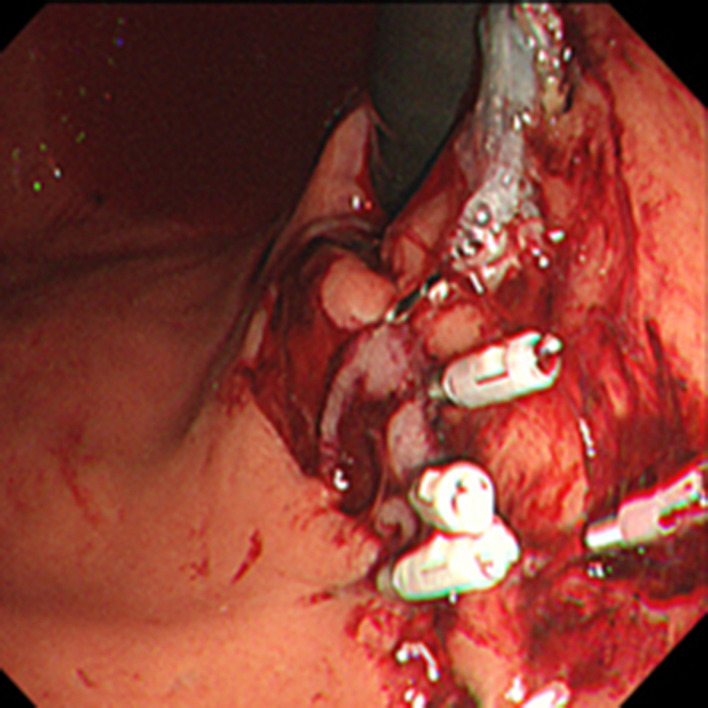
Repairment of perforation with clips.

Endoclips were the preferred treatment modality for 37 (84.1%) of the 44 patients (Figure 5-8). One patient with entry dehiscence was managed with OTSC. Four patients (9.1%) were treated with endo-loop suturing because their repair with endoclips was unsatisfactory. Fibrin glue was added to the treatment of 2 patients (4.5%) whose treatment with endoclips was insufficient.

The overall incidence of major AEs was 4.9%; 10 of the 206 patients experienced major AEs. The occurrence of major AEs in the MI group was significantly higher than that in the no-MI group. Patients with MI are significantly more vulnerable to major AE. Three patients (1.9%) experienced major AEs in the no-MI group, whereas 7 (15.9%) of the 44 patients in the MI group experienced major AEs (*P *< .05).

Age >50 years, previous POEM treatment, SMF, IPB, endoscopic diameter of 9.9 mm, and procedure duration of over 60 minutes were identified as independent predictors of MI during POEM in the univariate analysis (Table 5). When risk factors were adjusted, SMF, IPB, endoscopic diameter of 9.9 mm, and procedure duration of over 60 minutes were independent predictors of MI (Table 5).

## Discussion

Most studies that aimed to evaluate the safety of POEM have reported a low incidence rate of serious AE. One of the first classifications of AEs related to POEM was suggested by an international survey study (IPOEMS),^9^ which highlighted 2 main classes of AEs: minor and major AEs. One of the earliest and largest case series did not use such a classification and reported a rate of 3.2%; the AEs reported consisted mostly of serious but non-fatal AEs.

A recent study on AE related to POEM proposed that POEM is a unique endoscopic procedure in which some events cannot be evaluated as AE, especially insufflation-related events. In addition to this argument, the authors had developed a new grading system for AEs related to POEM. That study proposed a classification system that depends on the time of occurrence and severity.^6^ Major AEs that were listed were similar to those established by IPOEMS. Furthermore, they included MI patterns.

The findings of the present study were consistent with those of some studies that reported MI incidences. A study published by Wang et al^7^ comprehensively evaluated the clinical and endoscopic predictors of MI. Their reported 21.4% MI incidence rate is in accordance with the present study’s 21.4 % MI incidence rate. A recent study revealed a similar rate of MI incidence, with a 24% rate of minor mucosal defect, and only 1 patient was referred for surgery because of delayed cardia perforation.^10^ In contrast, an international multicenter study reported a lower rate of MI occurrence when compared with the aforementioned studies. In this study, 51 (2.8%) patients experienced inadvertent mucosectomy.^4^ A wide range of MI incidences may result from the definition of MI. It is possible that scald lesions were not marked as MI during some of the retrospective image analyses of the previous studies.

The most common injury pattern occurring during POEM was scald lesions, followed by SELs and perforations. Wang et al^7^ first described this type of injury pattern classification, and found that SEL was the most common type of injury pattern, followed by scald lesions, though the difference was not significant. Of the MI patients, 46.8% had SELs, whereas 39.8% of MI patients had scald lesions. Perforations were the least-seen type of injury pattern in the present study as well as in the Wang et al^7^ study.

Intra-procedural interventions included the use of endoclips, endoscopic stents, fibrin glue, OTSC, and endo-loop. There is no precise recommendation for MI in the guidelines to date, but we repaired all 3 types of injuries. In the Wang et al^7^ study, all of the patients with MI underwent any one of the available interventions for MI. Only a minority of patients received combined treatment modalities with fibrin glue and/or endo-loops. Otherwise, the clips achieved satisfactory results for the repair protocol. Another option is that scald lesions may be left without any intervention and followed up closely. Some endoscopists may prefer not to perform any intervention for scald lesions as they may not cause serious problems that affect POEM outcomes.^4,6^ Post-procedural surveillance is needed to prove this hypothesis. We suggest that MI classification based on injury patterns and a classification system based on the difficulty of intervention needed to repair MI can be used for further studies.^7^

Most of the previous studies were focused on the predictors of major AEs, or included all AEs rather than making a classification such as “major” or “minor.”^2^ Although the incidence rate of POEM-related AEs is low, these AEs may be serious and can lead to mortality or morbidity. An MI can be classified as major or minor AE depending on whether it can be managed without any intraoperative intervention or whether it needs to be repaired endoscopically or surgically. Third-space endoscopy depends on reaching the submucosal area and intra-abdominal space via mucosal incision; thus, protecting the integrity of the GI tract (GIS) mucosa is important to prevent contamination of the mediastinum or peritoneum. Any unintentional MI must be managed appropriately. Appropriate closure of the mucosal incision, which is the first step of POEM, is another example of protecting mucosal integrity. Severe MI, such as perforation or inappropriate closure of the mucosal incision, causes leakage of the GIS secretion into the submucosal tunnel and mediastinum.

A submocosal fibrosis is an esophageal feature that brings technical challenges, with reference to difficulty in separating the mucosal and muscular layers and no lifting sign after saline injection during the creation of the submucosal tunnel. Previous studies reported that SMF can result in an ineffective submucosal tunnel whose consequences increase complications and lead to the premature termination of the procedure.^11^ The fibrotic area secondary to inflammation as a result of a previous treatment, or a an SE, is an important obstacle to the procedure because it precludes the creation of the submucosal tunnel.

In the present study, the patients with SMF had a risk of developing MI that was 8.3 times that of the patients without SMF. MI occurrence may be a result of the poor orientation of the knife and thermal injury caused by the knife, especially in narrow spaces such as the cardia. Wang et al^7^ found results similar to our findings and reported an increased risk of MI occurrence in patients with SMF associated with an aOR of 4.5.

Previous studies have reported techniques to predict the presence of SMF. Feng et al^12^ conducted a study that aimed to find an association between the endoscopic classification of the esophageal mucosa in achalasia and SMF. It was shown that the appearance of the esophageal mucosa can predict SMF. Accordingly, a granular mucosa without obvious vascular texture, pachyntic, striated mucosa, and mucosa with ulcers and/or scars can predict SMF.

Another study that aimed to apply a scoring system to predict the difficulty of the POEM procedure included SMF in this scoring system.^13^

In order to predict SMF, we measured the thickness of the muscularis propria at the EGJ with endosonography before the POEM procedure for all patients. If the patient’s MP was over 3 mm, and/or the patient had a history of POEM treatment, the patient was considered to have a possibility of having SMF. The procedures of the patients who were suspected to have SMF were performed more carefully and slowly. The techniques that were used to avoid MI can be found in Table 6.

SMF, SE, and previous treatments are associated with each other. SE and previous treatments may accelerate an inflammation process in the submucosa that can cause SMF. The findings of the present study highlighted that SMF is an independent risk factor for MI after adjusting for other confounding factors. This finding may be interpreted as indicating that whatever the reason is, the prediction of SMF is important to avoid unexpected complications and procedural failure.

The aOR value of SE was found to be 3.6, but it did not reach significance according to our study findings. Although Wang et al^7^ reported that the SE is an independent predictor of MI, its aOR was 1.4 times lower than the other risk factors.

Symptom recurrence after a procedure is a sign of a complex disease when compared with other individuals. With the emergence of POEM as a new technique, patients with previous treatment failures have become eligible candidates for this novel technique. In addition to the complexity of the disease, SMF due to previous treatment is a candidate risk factor that can affect the success and safety of POEM. The present study did not find an association between the previous treatments and MI. The rate of MI incidence in patients with a previous history of BTI was significantly higher than that in the other patients. There was no significant difference between the no-MI and MI groups in terms of the history of previous POEM and HM. Our study findings are contradictory to the those of the Wang et al^7^ study, which revealed that patients with a history of POEM and HM were more vulnerable to MI. According to a multicenter study, half of the patients who experienced AE had undergone previous treatments, but that study did not provide statistical value regarding the comparison between patients with and without AE.^4^ Another study that aimed to evaluate AEs related to POEM found no association between previous treatment history and any AE.^10^ This difference in results may be explained by the number of patients who underwent POEM or HM being lower in our study than in the Wang et al^7^ study.

The use of endoscopes with an outer diameter of 9.9 mm was found to be an independent predictor of MI. The accessories that were used in POEM procedures were changed, from endoscopes with an outer diameter of 9.9 mm to those with an outer diameter of 8.9 mm. This change was made in December 2018 in accordance with the endoscopists’ observations that a larger outer diameter may influence the AEs related to POEM. Hence, this parameter was included in the analysis. To the best of our knowledge, this study is the first to indicate that a larger endoscope diameter can increase the risk of MI during POEM. It can be assumed that a smaller-caliber endoscope will cause less harm to the mucosa of the GIS than the larger ones. As third-space endoscopy is performed in a narrow area, smaller-caliber-sized endoscopes will benefit in terms of movement and working. The disadvantage of these techniques is the smaller working channel. This challenge can be overcome as devices suitable for third-space endoscopy are developed.

The present study showed that IPB occurred more often in individuals with MI, and was shown to be a significant predictor of MI occurrence; this can be attributed to an obscure view in a narrow submucosal area due to bleeding. Another possible reason for this association is the use of coagulation forceps for hemostasis on the mucosal side. Previous studies have shown that IPB is one of the most common AEs occurring during POEM. One study revealed that 9.5% of patients who underwent POEM experienced intense bleeding requiring prolonged hemostasis, and only 2 patients had delayed submucosal hematoma.^10^ The present study showed that 40 (19.4%) patients experienced IPB. This study is the first to formally report the association between self-limiting IPB and MI occurrence.

A procedure duration of more than 60 minutes was found to be an independent predictor of MI occurrence, but the association was weak (aOR = 1.016). The long procedure duration can be attributed to various factors. The learning-curve effect, SMF, IPB, and SE can be linked to a longer procedure time. Although procedure time was an independent risk factor, after adjusting for these risk factors, the aOR was still very low. We believe that this finding was incidental; hence, it must be interpreted cautiously in clinical practice.

The retrospective design is the main limitation of the present study, and it included only single-center data. Secondly; MI was assessed on images taken during the POEM procedure retrospectively; not visually during POEM. Another limitation is that some predictors that were mentioned by previous studies were not included in the analysis in thee present study. Mucosal edema was first defined by Zhang et al^2^ and accepted as a risk factor for MI. The present study did not include this variable as a risk factor in our patient work-up. If the patient’s initial assessment endoscopy showed mucosal edema, the POEM procedure was postponed until the unhealthy esophageal mucosa became normal mucosa. Physician-related factors were also not included in the analysis. All of the procedures were performed by an experienced endoscopist, and thus present study did not include the learning-curve effect. Previous studies that aimed to identify the predictors of AE were conducted as multicenter studies or involved more patients and more endoscopists in the education process of POEM. Large prospective multicenter studies must be conducted to provide more evidence about predictors of MI.

In conclusion, the present study revealed that IPB, a larger endoscope outer-diameter size, and SMF are strong risk factors for MI. Since MI is a significant event encountered during POEM, endoscopists should pay more attention to the risk factors associated with it to avoid adverse events.

## Figures and Tables

**Table 1.** Definitions Used in Manuscript

**Definitions**	
Patient-related data	Patients’ demographic data (age, sex) Esophagus motility disorder types (including Achalasia type) Disease duration Previous treatments (Heller myotomy, POEM, medical therapies, balloon dilatation, and Botulinum toxin injection) Esophagus features (sigmoid esophagus, submucosal fibrosis) Eckard score IRP measurement using a high-resolution manometer CSA measurements using a 40-mmL EndoFlip™ DI measurements using EndoFlip™
Procedure-related data	Intra-procedural bleeding Tunnel length Myotomy length Direction of myotomy Endoscope outer diameter size Procedure time Adverse event
Major adverse event	Insufflation-related AEs leading to hemodynamic instability and/or premature termination of the procedure IPB or delayed bleeding requiring transfusion, re-intervention, or hemodynamic instability Mucosal injury prolonging hospital stay Pneumonia Mediastinitis
Classification of mucosal injury	*Scald* (whitish or reddish lesions that do not expose the submucosal layer) *Submucosal exposure lesion* (lesions that expose the submucosal layer ) *Perforation* (full-thickness loss of the mucosa and submucosa that allows direct communication of the mediastinum and gastrointestinal lumen if it is not repaired appropriately.

POEM, Per oral endoscopic myotomy; IRP, integrated relaxation pressure; CSA, cross-sectional area, EndoFlip, endoscopic functional imaging probe; DI, distensibility index; AE, adverse event, IPB, intra-procedural bleeding.

**Table 2.** Equipment Used in POEM Procedure

**Equipment**	**Brand Name**	**Models**
Processor	Olympus Co., Tokyo, Japan	GIF H29OZ 9.9 mm GIF H290 8.9 mm
Electrosurgical unit	VIO300D; Erbe, Germany	Mucosal entry; EndoCut mode 50W, effect 2 Submucosal tunnel; Spray coagulation, 50W, effect 2 Myotomy; Endocut mode 50W, effect 2
Knives	Olympus Medical Systems, Tokyo, Japan	Mucosal entry; Dual Knive KD-650L Myotomy; Hook knife KD-620LR
Coagulation	Olympus Medical Systems, Tokyo, Japan	Coagulation forceps; Coagrasper FD-410LR

**Table 3.** Characteristics of Eligible Patients and Procedure Details

**Variables** No. of patients	**Total** 206	**No. of MI** 162	**MI** 44	***P***
Patient factor				
Age, years, mean (SD)	39.1 (14.6)	39.1 (14.5)		.817†
Sex				.558¶
Male	98 (47.6%)	77 (47.5%)	21 (47.7%)	
Female	108 (52.4%)	85 (52.5%)	23 (52.3%)	
Disease duration, years		5.9 (8.0)	6.5 (8.1)	.644†
Prior intervention				.043¶
None	119 (57.8%)	97 (59.9%)	22 (50%)	
Calcium channel blocker	13 (6.3%)	10 (6.2%)	3 (6.8%)	
Balloon dilatation	33 (16%)	30 (18.5%)	3 (6.8%)	
Botulinum toxin injection	25 (12.1%)	15 (9.3%)	10 (22.7%)	
Heller myotomy	9 (4.4%)	6 (3.7%)	3 (6.8%)	
POEM	7 (3.4%)	4 (2.5%)	3 (6.8%)	
Eckardt score		6.2 (2.8)	6.9 (2.5)	.14†
IRP score		27.9 (19.9)	22.3 (15.1)	.047†
Endoflip (40 mmL)				
CSA		93.6 (87.8)	108.1 (67.6)	.397†
DI		3.0 (3.0)	3.2 (2.4)	.793†
Achalasia type				.734¶
Type 1	70 (34.3%)	56 (34.8%)	14 (32.6%)	
Type 2	85 (41.7%)	66 (41.0%)	19 (44.2%)	
Type 3	14 (6.9%)	10 (6.2%)	4 (9.3%)	
Variant	3 (1.5%)	2 (1.2%)	1 (2.3%)	
DES	4 (2.0%)	4 (2.5%)	0 (0.0%)	
Jackhammer	4 (2.0%)	2 (1.2%)	2 (4.7%)	
EGJOO	22 (10.8%)	19 (11.8%)	3 (7.0%)	
Ineffective motility disorder	1 (0.5%)	1 (0.6%)	0 (0.0%)	
Nutcracker esophagus	1 (0.5%)	1(0.6%)	0 (0.0%)	
Esophageal features				
Sigmoid esophagus	22 (10.7%)	14 (8.6%)	8 (18.2%)	.067¶
Submucosal fibrosis	9 (4.4%)	2 (1.2%)	7 (15.9%)	<.001¶
Procedure factor				
Intraprocedure bleeding	40(19.4%)	17(10.5%)	23 (52.3%)	.04¶
Scope outer diameter				.014¶
9.9 mm	176(85.4%)	144(88.9%)	32 (72.7%)	
8.8 mm	30(14.6%)	18(11.1%)	12 (27.3%)	
Full-thickness myotomy		150(86.4%)	40 (90.9%)	.76¶
Tunnel length, cm		11.8 (2.4)	12.1 (2.5)	.391¶
Myotomy length, cm		3.6 (1.7)	3.6 (1.8)	.77¶
Procedure duration		66.5 (28.7)	88.9 (35.3)	<.001¶

^*^ *P* value calculated by using the Kruskal–Wallis rank-sum test.^ †^ *P* value calculated by using the one-way ANOVA.^ ¶^ *P* value calculated by using the Fisher’s exact test, Numbers in bold indicate a significant difference (*P* < .05). Y, years; SD, standard deviation; IRP, integrated relaxation pressure; CSA, cross-sectional area; DI, distensibility index; POEM, peroral endoscopic myotomy; DES, diffuse esophageal spasm; EGJOO, esophagogastric junction outflow obstructrion; cm, cen­timeter; mm, milimeter.

**Table 5.** Predictors of Mucosal Injury.

		**Univariate**			**Multivariate**		
		**OR**	**95% Cl**	***P***	**aOR**	**95% Cl**	***P***
Patients factor							
Age	≥ 50 years	2.065	[1.038-4.108]	0.039	1.012	[0.983-1.042]	0.414
Sex	Male	1.054	[0.538-2.065]	0.879			
Disease duration	≥ 4 years	1.377	[0.702-2.701]	0.352			
Past medical history							
Calcium channel blocker		1.579	[0.397-6.278]	0.517			
Balloon dilatation		0.702	[0.148-3.322]	0.655			
Botulinum toxin injection		1.754	[0.737-4.178]	0.204			
Heller myotomy		2.632	[0.605-11.447]	0.193			
POEM		13.158	[2.376-72.867]	0.003	5.634	[0.833-38.119]	0.076
Eckardt score	≥8	1.385	[0.699-2.744]	0.351			
IRP score	≥20	0.763	[0.386-1.510]	0.438			
Esophageal features	Sigmoid esophagus	2.349	[0.916-6.026]	0.076	3.271	[0.930-11.503]	.065
	Submucosal fibrosis	15.135	[3.020-75.842]	0.001	8.332	[1.318-52.667]	.024
Procedure factor							
Intra-procedure bleeding		9.342	[4.299-20.300]	<0.001	14.29	[5.589-36.535]	<.001
Endoscopic Caliber	Large diameter 9.9 mm	3	[1.315-6.844]	0.009	4.389	[1.523-12.651]	.006
Full-thickness myotomy		1.282	[0.658-2.498]	0.466			
Direction of myotomy	Posterior myotomy	0.833	[0.427-1.626]	0.593			
Tunnel length	≥ 13 cm	1.862	[0.951-3.646]	0.07	1.101	[0.925-1.310]	.279
Myotomy length	≥ 10 cm	1.239	[0.627-2.450]	0.537			
Procedure duration	≥ 60 minutes	2.743	[1.237-6.082]	0.013	1.016	[1.001-1.032]	.034

OR, odds ratio; aOR, adjustable odds ratio; y, years; POEM, peroral endoscopic myotomy; IRP, integrated relaxation pressure.

**Table 4.** Mucosal Injury Characteristics and Treatment Details

**Description**	**Total (%)**
Number of cases	44 (21.4)
Location	
Esophagus	8 (18.20)
Cardia	36 (81.80)
Injury patterns	
Mucosal scald	21 (47.7)
Submucosa exposure	15 (34.1)
Perforation	8 (18.2)
Closure approach (number, %)	
Endoclips	37 (84.1)
Clip count, median	6
Endo-loop and clips	4 (9.1)
Fibrin glue	2 (4.5)
Over-the-scope clip	1 (2.3)
Diameter, mm, median	13

mm, milimeter.

**Table 6.** Techniques Used to Avoid Mucosal Injury

Techniques used to avoid mucosal injury	Preprocedure	Measurement of thickness of muscularis propria of EGJ during preprocedure EUS. ≥ 3 mm indicates SMF Defining mucosal edema and postponing the procedure until the mucosa is healed	During procedure	Keeping the knife orientation close to the muscular layer Adequate use of injections during tunnel creation Creating a wide tunnel before myotomy Slowing down at EGJ to find palisade veins. Checking the direction by pulling out the scope at regular intervals Keeping the tunnel and myotomy planes linear Maintaining the myotomy line in the middle of the tunnel

EGJ, esophagogastricjunction; EUS, endosonography; SMF, submucosal fibrosis.
